# New Deposit of Mordenite–Clinoptilolite in the Eastern Region of Cuba: Uses as Pozzolans

**DOI:** 10.3390/molecules26154676

**Published:** 2021-08-02

**Authors:** Jorge Luis Costafreda, Domingo Alfonso Martín

**Affiliations:** Escuela Técnica Superior de Ingenieros de Minas y Energía, Universidad Politécnica de Madrid, C/Ríos Rosas, 21, 28003 Madrid, Spain; domingoalfonso.martin@upm.es

**Keywords:** mordenite–clinoptilolite, smectite, altered volcanic glass, pozzolanic reactivity

## Abstract

This work describes the newly discovered zeolites in the eastern region of Cuba. In the researched area, there have been no previous studies of natural zeolite exploration. Therefore, the results shown here are new. The main object of this research is to analyse five samples of zeolites and demonstrate their pozzolanic capacity and the possibility of their usage in the industrial manufacturing of pozzolanic cements. The study of the samples was performed by X-ray diffraction (XRD), X-ray fluorescence (XRF) and scanning electron microscopy (SEM). A chemical analysis (CAQ) to determine the quality of the samples as pozzolans was performed, by determining the total SiO_2_, reactive SiO_2_, total CaO, reactive CaO, Al_2_O_3_, MgO and the insoluble residue (I.R.). Lastly, an eight-day pozzolanicity analysis (PA) was carried out to determine the pozzolanic reactivity of the samples. The results obtained by XRD, XRF and SEM established that the researched zeolite samples have two main zeolitic phases: mordenite and clinoptilolite. Altered volcanic glass, quartz and smectite (montmorillonite) are the secondary phases. The results of the chemical quality analysis (CAQ) showed that the samples contain a considerable amount of reactive SiO_2_ and reactive CaO, as well as a low content of insoluble residue, which reinforces their properties as pozzolans. The results of the pozzolanicity analysis (PA) concluded that the analysed samples actively react with Ca(OH)_2_ after eight days. Based on all the results mentioned above, it is established that both mordenite and clinoptilolite behave like pozzolans and can be recommended for the manufacture of pozzolanic cements, which have more effective properties than Portland cement, in terms of physical, chemical and mechanical strength, low heat of hydration, resistance to sulphates, low CO_2_ emissions to the atmosphere and negligible impacts on the environment.

## 1. Introduction

**Figure 1:**
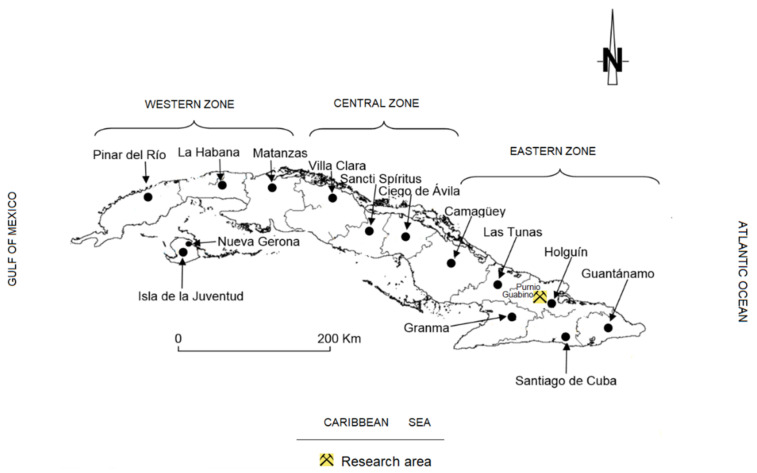
Location of the area of study.

Several magmatic cycles formed the volcanic arc of Cuba during the Cretaceous. The erosive process created large deposits of volcano-sedimentary materials in the deposition basins; many of these materials were enriched with volcanic glass that was gradually altered by meteoric waters and hydrothermal processes, forming large deposits of zeolites in many places in Cuba. This paper presents the results of the research on the characterisation of new indications of natural zeolites of the eastern part of the Republic of Cuba, in the north-west of Holguín (Figure 1). Knowledge of zeolites in Eastern Cuba is extensive, although not all deposits have been definitively discovered or studied. Zeolites represent an important link to the economy of this country, so, based on this fact, the following is a brief description of the use of zeolites in Cuba.

Zeolites were first described in the province of Holguín in 1971 by geologists of the Institute of Geology and Paleontology (IGP) of La Habana (Cuba) [1,2]. Subsequently, a group of Cuban and Hungarian geologists carried out geological reconnaissance work on the locality of Loma Blanca, in San Andrés [3]. Researchers from the University of Moa and the Polytechnic University of Madrid also conducted a surveys for zeolites in the Loma Blanca deposit [4,5,6]. The result of their research allowed the opening of a mine, which is at present being operated.

Currently, zeolites are widely used in various fields of the Cuban economy. The clinoptilolite is used to promote growth in plants and is a very valuable fertiliser [7]. It is used as a molecular sieve and as a means of filtering [4]. Cuban zeolites are used as food additives in animal diets and in the treatment and prevention of certain illnesses in poultry farms [8]. It is in great demand in construction as a pozzolanic material that offers good physical–chemical properties to cement [9]. These zeolites have been successfully characterised for the manufacturing and development of nanostructured materials [10]. The pozzolanic cements made with mordenite–clinoptilolite possess mechanical strength superior to 50 MPa for up to 28 days [11]. The mordenite–clinoptilolite from the Loma Blanca deposit is used in pharmaceutical products due to its properties of irreversible acquisition of histamine under both acidic (pH 3.5) and neutral (pH 7) conditions [12]. It is used in the processes of lead absorption and in the decontamination of waters [13]. Other research indicates that these zeolites are efficient in catalytic processes for their thermodynamic and thermophysical properties [14]. Buenaño et al. [15] carried out a study of cation exchange with zeolite from the Tasajeras deposit (Cuba) as an alternative to manage acidic water, using a breakthrough curve methodology. Cháves et al. [16] have used zeolites from the Loma Blanca deposit as a cation exchanger to capture rare-earth elements in the debris of depleted metal deposits. This zeolite (clinoptilolite) has a high cation exchange capacity, which provoked an increase in the capture of rare-earth elements until the breaking point was reached. Cuban zeolite was widely used in urban agriculture during the world socialist block collapse [6], where it was used as a substrate in zeoponics for the provision of greens and vegetables to the populace. These zeolites are effective in the formulation of medicines such as petrolatum and squalene for the treatment of mycosis [17]. In the latest research, clinoptilolite was used from the Tasajeras deposit in the development of reactive permeable walls to extract inorganic contaminants from subterranean water [18]. The study by Cerri et al. [19] demonstrated that mordenite–clinoptilolite from the Loma Blanca deposit, Holguín, in its sodium form (M-Na) prepared by cation exchange, is able to inhibit the growth of Helicobacter Pylori bacteria. At the present moment, the deposit of Loma Blanca (San Andrés) is being mined. It is a concession of the Geological Unit of San Andrés, a subsidiary of the Geomining Company of the Orient (Santiago de Cuba). The greater part of the production of zeolite (mordenite-clinoptilolite) is exported to Central America and South America. The national production of zeolite from the Republic of Cuba amounted to 53,000 tons in 2020 [20]. The other part of this plant’s production, together with the plant deposit of Tasajeras, in Villa Clara (central Cuba), is used in various national industries.

The present work had two main phases. In the first phase, a mineralogical, petrological and chemical study of zeolites found in an unexplored area in the eastern region of Cuba was carried out; in the second phase, their technological qualities were determined in order to establish their properties as pozzolans. The studies carried out in both phases considered three fundamental questions: the geological nature of the deposit, the pozzolanic properties of these zeolites and the possibility of their use as a raw material in the manufacture of pozzolanic cements.

## 2. Geological Setting

The area of study is within the Loma Blanca Formation from the Lower Cretaceous Aptian to the Upper Cretaceous Campanian [3]. It is divided into two geological units, the Normal Series of volcanogenic–sedimentary composition, formed during the subduction of the oceanic plate with the arc formation of volcanic islands, and a Final Series, during the tectonic destruction of the volcanic arc accompanied by sintectonic magmatism with a formation of subvolcanic bodies [21]. The materials are tuff, clays and zeolites of medium and acidic composition, which are sedimented in deep or relatively deep pelagic waters. In the upper part, acidic and sedimentary rocks were formed. The marl and limestones are mixed with sediments of volcanic origin of an andesitic and dacitic composition, with the presence of radiolarians [3,22,23].

## 3. Results and Discussion

### 3.1. X-ray Diffraction (XRD)

**Figure 2:**
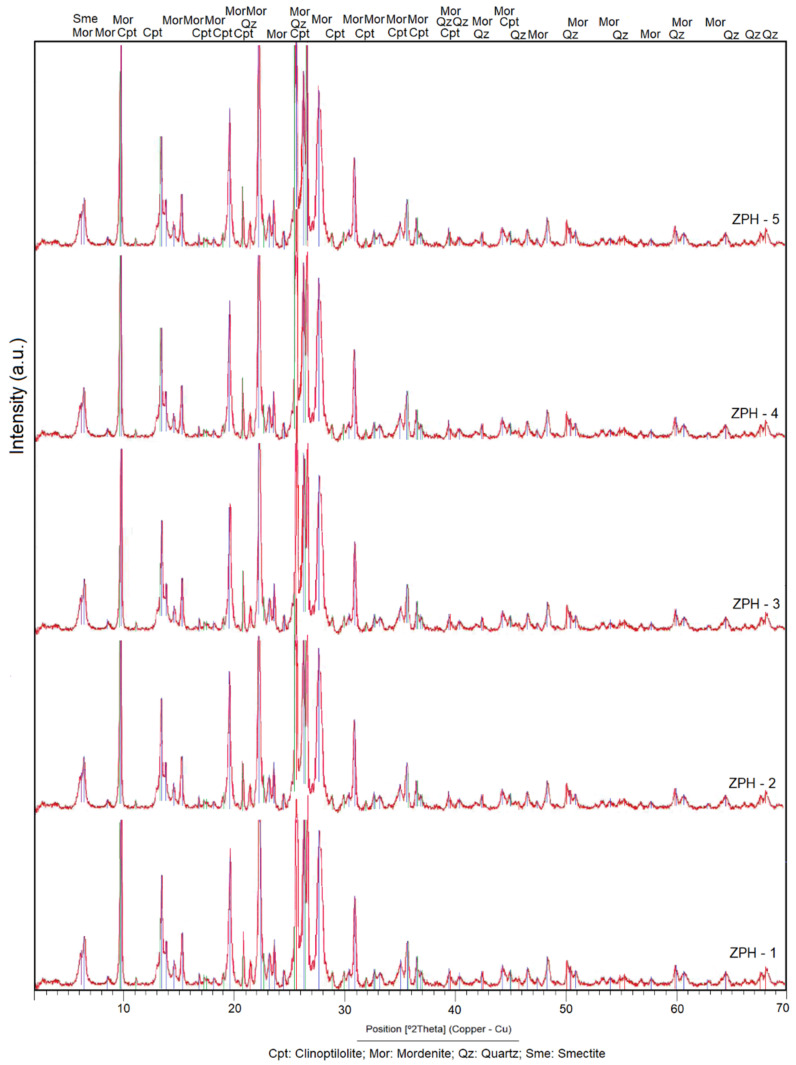
X-ray diffraction patterns of the natural mordenite–clinoptilolite from the research area.

The results obtained by XRD established that the researched zeolite samples have two main zeolitic phases, mordenite and clinoptilolite, which is evident in the X-ray diffraction patterns presented in Figure 2. Altered volcanic glass, quartz and smectite (montmorillonite) are the secondary phases. According to Figure 2, the samples have a high concentration of zeolitic ore.

The peak of maximum intensity (Imax = 100%) of the mordenite is located at 2θ = 26.65 (Figure 2) (the term 2θ is equivalent to the position °2 Theta). Other peaks stand out at the angular positions of 2θ from 9.75 to 35.68. The remaining peaks are the lowest in intensity.

In clinoptilolite, the peak of greater intensity (Imax = 100%) is located at the angular position 2θ = 9.88 and is overlapping with a mordenite peak. A second peak of clinoptilolite of appreciable intensity is seen in 2θ = 11.19, with an intensity equal to 40%. Other peaks with less intensity appear at angular positions 13.06 to 32.01, with intensities varying between 14% and 48.2%.

Above 2θ = 45.0, the peaks of clinoptilolite are masked by mordenite, which is interpreted as an isomorphic substitution between the two minerals, possibly caused by a variation in the alkalinity/acidity of the geological environment [3,21,24]. The chemical composition data shown in Table 1 indicate a calc-alkaline composition of the analysed samples [21].

**Figure 3:**
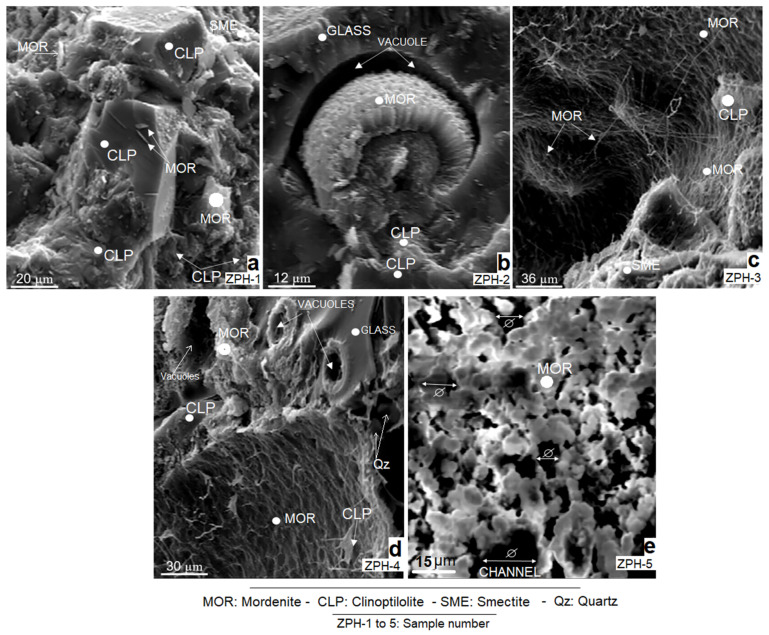
SEM micrographs of natural mordenite-clinoptilolite samples: (**a**) sample ZPH-1; (**b**) sample ZPH-2; (**c**) sample ZPH-3; (**d**) sample ZPH-4, and (**e**) sample ZPH-5.

Quartz has its peak of maximum intensity (Imax = 100%) shifted towards the angular position 2θ = 26.54. Peaks with medium intensity are observed distributed at positions 2θ from 36.45 to 59.87. Quartz peaks with even lower intensity have not been taken into consideration in the discussion, although they can be seen in Figure 2. The presence of quartz in the analysed samples permitted us to infer that the zeolites discovered were formed from the hydrothermal alteration of acidic and intermediate rocks such as rhyolites, dacites and andesites. The altered volcanic glass, as shown in Figure 3d, confirms this fact, as the acid and intermediate rocks contain more glass than the basic rocks.

The peak of maximum intensity of the smectite (montmorillonite) is located at 2θ = 5.89, but it is hardly perceptible. The peaks that identify the smectite at positions 2θ = 19.91 to 2θ = 54.31 are not easily seen or do not exist; this means that the process of smectisation of the glass barely developed. It follows that the devitrification of the volcanic glass, affected by the hydrothermal processes, directly produced the mordenite and clinoptilolite phases, without the formation of smectite on a large scale. As seen in Table 1, the alkaline compounds of K_2_O and Na_2_O have higher values than MgO; this fact explains why the mordenite–clinoptilolite phase was developed more than the smectite phase (montmorillonite). A similar approach has been formulated in the last decade by Costafreda [24]. The mineralogical composition of the samples studied significantly favours pozzolanic characteristics, which reinforces the objectives in this research.

### 3.2. Scanning Electron Microscopy (SEM)

The study carried out by scanning electron microscopy (SEM) confirms the majority of mordenite and clinoptilolite and, to a lesser degree, smectite (montmorillonite) in all analysed samples. It is observed that the formation of these three mineral species depends on the degree of volcanic glass alteration, as is confirmed in Figure 3b–d.

Figure 3a corresponds to sample ZPH-1, which shows aggregates of well-developed idiomorphic crystals of clinoptilolite (Clp). These crystals are surrounded by compact microcrystalline aggregates of clinoptilolite that grow in very tight masses. The presence of smectite (Sme) is extremely limited, as discussed in Section 3.1; it forms small irregular hypocrystalline masses in the upper right of the microphotograph. Mordenite (Mor) forms small idiomorphic crystals that grow at the expense of clinoptilolite.

Figure 3b (sample ZPH-2) shows an open vacuole (Vc) formed in the devitrified glass. Inside this vacuole, mordenite crystals grow profusely, with marked idiomorphic traits and with a reniform, nodular and radial habit. The new crystals have formed around a botryoidal nucleus of older mordenite aggregates. The textures observed indicate two processes of zeolitic mineralisation: an earlier and a later one.

Figure 3c (sample ZPH-3) depicts white mordenite with an acicular, filiform, fibrous and reticular habit; occasionally, it resembles bacilli. It has a strong spatial and possibly genetic relationship with smectite. The smectite forms small masses and irregular aggregates. Its abundance is noticeably lower than mordenite. At the bottom of the microphotograph is a fragment of volcanic glass practically replaced by mordenite and smectite.

In Figure 3d (sample ZPH-4), fibrous and reticular mordenite persists, although it also forms irregular microcrystalline aggregates near the edges of the vacuoles formed in the volcanic glass. It is clear that both mordenite and smectite have been formed at the expense of glass. Clinoptilolite has a limited presence in this sample.

Figure 3e (sample ZPH-5) depicts a single mineral phase of mordenite. It forms interconnected crystalline aggregates and develops annular structures. It has a reticular, porous and annular structure. The entrances of the channels are observed to have wide openings.

Mordenite and clinoptilolite are present in practically all the samples analysed. They have vast crystalline development. This was also observed in Figure 2; therefore, the aspects discussed in both sections are confirmed.

### 3.3. X-ray Fluorescence (XRF)

Analysis by X-ray fluorescence (XRF) shows the chemical composition of the samples studied, which is of the utmost importance for this research, since the major components have been identified, such as SiO_2_ and Al_2_O_3_, that determine the pozzolanic capacity of the samples.

The contents of the major components are homogeneous in the analysed samples. The silica shows the highest values, which vary between 64.20% and 65.02% (Table 1). The Al_2_O_3_ shows content from 11.30 to 13.25%. The CaO is between 1.95% and 4.09%. The K_2_O has content from 1.65 to 2.33%. The Na_2_O varies between 0.22% and 1.34%. The loss on ignition (LOI) has high values, between 12.11% and 14.53%. The content of Fe_2_O_3_ is between 1.32% and 1.83%, while the MgO ranges from 0.25 to 0.74%. The Si/Al ratio varies from 4.3 to 5, where the lowest value corresponds to clinoptilolite, and the highest to mordenite.

**Figure 4:**
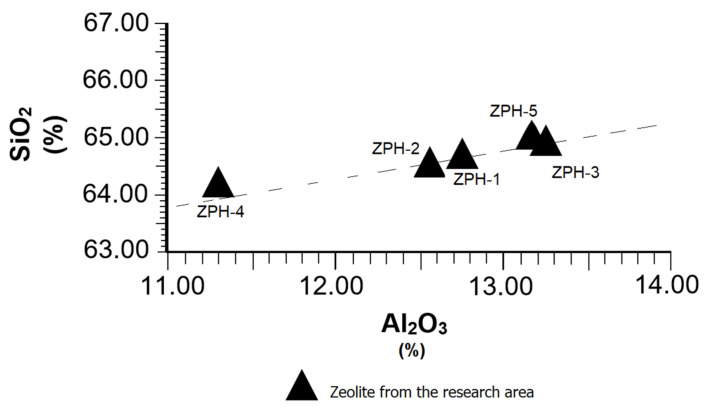
Behaviour of the SiO_2_ vs. Al_2_O_3_ in the mordenite–clinoptilolite.

Figure 4 specifically represents the SiO_2_ and Al_2_O_3_ relation, according to some data extracted from Table 1. From the average values of both compounds, a SiO_2_:Al_2_O_3_ = 5.13 ratio was calculated, which is suitable in natural pozzolans of zeolitic type. This high ratio makes Al_2_O_3_ behave as an acid compound that reinforces the action of SiO_2_ in the development of pozzolanic reactions. The results of Figure 4 highlight the fact that the Al_2_O_3_ values never reach 16% in any case, which is favourable for producing pozzolanic cements, because an excess of alumina greater than 16% could reduce the mechanical strength of the cement [24]. In spite of the differences seen in some samples of individual contents of SiO_2_ and Al_2_O_3_, they behave normally, as can be seen in the trend line.

The high values of loss on ignition (LOI) are highlighted in Table 1. Observation shows that they vary between 12.11 and 14.53%, this is due to the cation exchange capacity (CEC), porosity, adsorption–absorption–desorption (AAD) properties and Si/Al ratio inherent in the mordenite and clinoptilolite investigated [6]. It also means that these zeolites have reversible behaviour; that is, they can reabsorb the ions, molecules and water that they lost during the heating process, an ideal property for the manufacture of pozzolanic cements, which is the key objective of this research.

The low values of MgO (Table 1) are suitable for the objectives of this research, because if it is used in the manufacture of pozzolanic cements, there will only be small amounts of this compound. It is well known that an excess of MgO will cause expansive effects on cements and building structures [24]. CaO has exceptionally low values when compared to ordinary Portland cement; this is favourable to this research as it will facilitate the pozzolanic reaction of mordenite and clinoptilolite with Ca(OH)_2_. The relation Si/(Al + Fe) indicates very close and balanced values (Table 1). The behaviour of the CaO, K_2_O and Na_2_O according to Table 1 confirms the calc-alkaline character of the deposits where mordenite and clinoptilolite occur, which coincides with the results reported by Kozák and Rózsa [21].

### 3.4. Results of the Chemical Analysis to Determine the Pozzolanic Quality of the Samples Investigated

The materials employed in the fabrication of pozzolanic cements must be rigorously tested to determine their quality, as the UNE-EN 196-2:2014 standard [25] indicates. The analyses were performed to determine the content of total SiO_2_, reactive SiO_2_, Fe_2_O_3_, Al_2_O_3_, total CaO, reactive CaO, MgO, insoluble residue (I.R.) and SO_3_, as indicated in Table 2. The UNE-EN 196-2:2014 standard [25] also establishes maximum limits of compositions to guarantee the quality of the pozzolanic cement, as can be observed in Table 2. The relation SiO_2_/(CaO + MgO) gives results superior to 3.5% and indicates that this pozzolanic cement is resistant to sulphates and seawater attack. This is of great importance if one bears in mind Cuba´s marine environment. On the other hand, the relation SiO_2_ + Al_2_O_3_ + Fe_2_O_3_ gives values superior to 70%, in accordance with the ASTM C 618-89 standard [26]. The content of reactive SiO_2_ with respect to the total SiO_2_ indicates the strong reactivity of this compound as well as the crystalline state of these zeolites.

Table 2 shows that reactive SiO_2_ in the samples varies within a narrow margin (60.28 to 61.20%); total SiO_2_ has a similar trend, indicated by a variation of a few tenths (64.98 to 64.08%). However, the samples ZPH-3, ZPH-5 and ZPH-2 stand out due to their contents of both compounds. All this shows that the samples studied have quite a similar chemical and mineralogical composition, as has been seen in the discussions in Section 3.1, Section 3.2 and Section 3.3.

**Figure 5:**
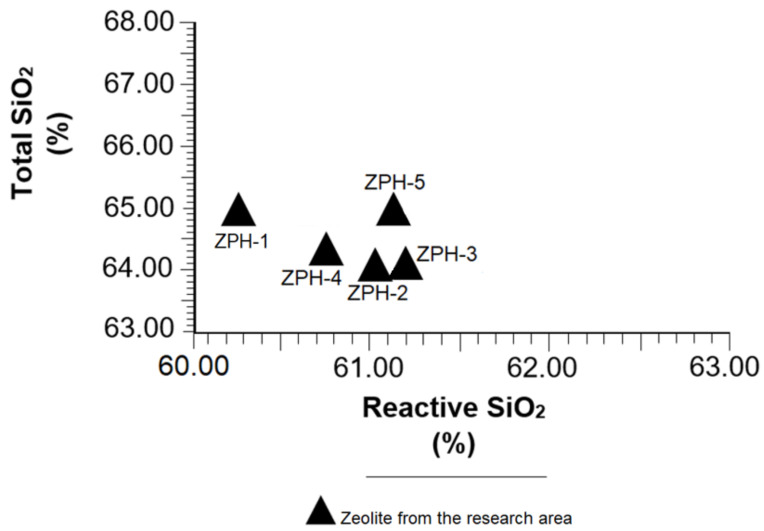
Variation of the total SiO_2_ vs. reactive SiO_2_ in the mordenite–clinoptilolite.

Another aspect to highlight is the ratio of total SiO_2_/reactive SiO_2_. Because of its importance, a graph has been created (Figure 5) to emphasise the behaviour of total SiO_2_ vs. reactive SiO_2_ in all samples analysed. It highlights the fact that reactive SiO_2_ is the soluble part of total SiO_2_, i.e., silica that is able to react with Ca(OH)_2_ in the hydraulic reaction system of cement; therefore, the greater the amount of reactive SiO_2_, the greater the pozzolanic reactivity. The average reactivity rate of soluble silica has been calculated at 96.3% with the help of the data shown in Table 2. This fact is verified in this research and is proven in Figure 5. The ASTM C 618-89 standard [26] states that all pozzolanic material must exceed 25% of reactive SiO_2_, an aspect very well represented in Table 2.

Table 2 shows exceptionally low SO_3_ values in all the samples analysed. The maximum value allowed by the UNE-EN 196-2:2014 standard [25] is 4%. It is necessary to argue that the absence of SO_3_ in the analysed samples is beneficial for the manufacture of clinker from pozzolanic cements, since zeolites have the ability to decrease the reactivity of C_3_A and avoid the abundant formation of ettringite, which has the tendency to expand and form fissures in cement structures. Because of this, the hydraulic reaction in the cement is slower, facilitating the total hydration of the bi-calcium silicates.

The ratio of total CaO vs. reactive CaO provides conclusive data for this research (Table 2). It should be noted that virtually all CaO can react, which prevents free CaO from remaining in the system. CaO is very harmful because of its tendency to expand and weaken cement structures.

The insoluble residue (I.R.) has values of 3.43 to 4.7%, remarkably close to the limit indicated by the UNE-EN 196-2:2014 standard [25]; however, this residue consists of non-soluble SiO_2_ found in the analysed samples. It is a highly crystalline SiO_2_ and very stable against the acids of the test; therefore, its presence in pozzolanic cements will be beneficial to the rheology of the cement.

In the case of Al_2_O_3_, the calculated values place this compound below 16% [25], as shown in Table 2. Al_2_O_3_ is of vital importance in pozzolanic reactions, as noted above in Section 3.3 and explained in Figure 4.

Finally, it is highlighted that the ratio of SiO_2_/(CaO + MgO): 14.50 to 24.0% and SiO_2_ + Al_2_O_3_ + Fe_2_O_3_: 76.91 to 79.60% significantly exceeds the minimum value imposed by the UNE-EN 196-2:2014 and ASTM C 618-89 standards [25,26].

### 3.5. Results of the Eight-Day Pozzolanicity Analysis to Determine the Pozzolanic Reactivity of the Samples

The pozzolanic capacity was determined by comparing the concentration of calcium hydroxide and the portion of calcium ions (expressed as calcium oxide) that saturates a solution with the same alkalinity, and also contains a hydrated cement; the test is considered positive when the calcium ion concentration in the solution is less than the saturation concentration [27]. Equations (8) and (9), used in this calculation, are explained in Section 4.2.5.

**Figure 6:**
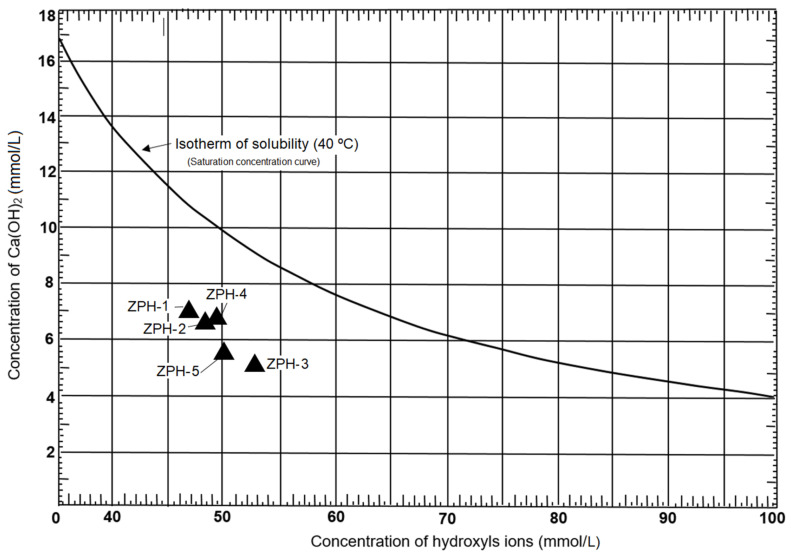
Pozzolanic reactivity of the mordenite–clinoptilolite.

The concentrations of hydroxyl ions and calcium oxide are represented in the shape of a triangle in Figure 6. As can be seen, this figure represents the concentration of saturation in calcium ions as a function of the concentration of hydroxyl ions in a solution at 40 °C. According to this diagram, the zeolites of the study area have a marked pozzolanic behaviour. This fact could be provoked by the high crystallinity of the silica in the zeolites analysed; similar results have been presented by Machiels et al. [28], Costafreda et al. [29] and Presa et al. [30]. The amorphous silica favours even more the pozzolanic reactivity, as has been established by Mirzahosseini and Riding [31]. Both the SiO_2_ and the Al_2_O_3_ (Table 2), are very important for the reaction of the mordenite–clinoptilolite with the Ca(OH)_2_ in solution; in this sense, Rosell-Lam et al. [11] reached a similar conclusion. This favours the fixation of free lime and the neutralisation of calcium hydroxide in solution, avoiding the later reaction with sulphates and chlorides [32]. The pozzolanic reactivity influences favourably the mechanical strength of the cement, mortars and concrete [33].

Although all samples have pozzolanic reactivity, some stand out more than others; for example, ZPH-3 is comparatively the most reactive, followed by ZPH-5. The hierarchical order of pozzolanic intensity is as follows: ZPH-2, ZPH-4 and ZPH-1. As mentioned above, this behaviour is due to the amount of reactive SiO_2_ present in every sample according to Table 2; it is evident that comparing these values with the position of the samples in Figure 6 shows a close coincidence. Note that total SiO_2_ is not considered in this comparison, even though its values are higher. In Section 3.4, it was explained that the total SiO_2_ contains a non-reactive portion, i.e., the most crystalline part considered as an insoluble residue (I.R.). Once the insoluble residue was separated, only the reactive SiO_2_ remained.

According to Table 2, practically all SiO_2_ can react; therefore, the values of reactive SiO_2_ of each sample have been averaged and it has been established that 96.3% of SiO_2_ contained in the analysed samples is soluble. The result of this calculation confirms the pozzolanic capacity of the researched zeolites.

## 4. Materials and Methods

### 4.1. Materials

**Figure 7:**
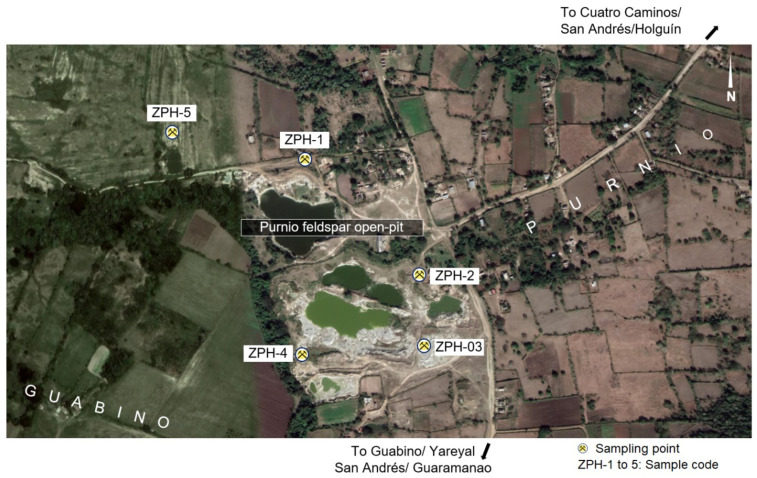
Location of the sampling places [34].

Five samples of zeolitic tuffs (HP-01, HP-02, HP-03, HP-04 and HP-05) were taken in various outcrops around a feldspar open pit to the north of the village of El Guabino (Figure 7). The samples were obtained by the method of channel sampling. The weight of each sample was 10 kg.

The samples were subjected to a conditioning process in order to obtain the appropriate sizes for the tests. To obtain an adequate particle size, crushing was carried out with a Wings jaw crusher, resulting in a maximum particle size of 3 cm. To reduce the size of the samples, further crushing was carried out with an Alas Jaw Crusher, which resulted in a maximum particle size of 3 cm. By means of a second Controls Jaw Crusher, the particle size was reduced to a maximum size of 1 cm. Finally, the material was ground using a Siebtechnik Vibratory Mill.

### 4.2. Methods

#### 4.2.1. X-ray Diffraction (XRD)

The analysis by XRD was performed using the crystal dust method (PTE-RX-004). The measurements were taken using the XPERT PRO MPD equipment (Almelo, The Netherlands) of PANalytical, composed of a copper tube (45 kV, 40 mA), a graphite monochromator and an automatic aperture. For data acquisition, X’Pert Data Collector 5.1 (5.1.0.156) of PANalytical was used. Moreover, the HighScore version software 3.0.4 (PANalytical) was also used, as well as the databases PDF-2 (ICDD) and CODJanuary2012 for the later analysis and interpretation of the data obtained.

#### 4.2.2. Scanning Electron Microscopy (SEM)

The morphological and textural features were observed using scanning electron microscopy (SEM). It was performed on a Philips-505 equipped (Amsterdam, The Netherlands) with an EDAX 9000 at Instituto de Catálisis y Petroleoquímica, CSIC (Spain). In these cases, the samples were observed without coating.

#### 4.2.3. X-ray Fluorescence (XRF)

The samples were analysed with Philips equipment (Amsterdam, The Netherlands), model PW 1404, with a collimator for reduction of the divergence angle of the X-rays. The intensity of the sample’s radiation oscillated between 10 and 100 kV. A monochromator was used to separate the measured radiation and to obtain an adequate wavelength. The samples were ground to 200 mesh. A quantity of 6–8 gm was mixed with 1.5 mL of elbaite. It was dried at room temperature for a duration of 5 min. The trial tablet was made with a diameter of 5 cm. One gm of sample was analysed to determine loss on ignition (LOI).

#### 4.2.4. Chemical Analysis to Determine the Quality of Zeolites as Pozzolans (CAQ)

This test was carried out following the requirements of the UNE-EN 196-2-2014 standard [25]. The primary objective was to determine several key compounds in the samples studied, because a small percentage can determine whether they are suitable for use as pozzolans in the manufacturing of pozzolanic cements. In order of hierarchical importance, the compounds detected were: total SiO_2_ (TS), reactive SiO_2_ (RS), total CaO (TC), reactive CaO (RC), MgO, Al_2_O_3_ and Fe_2_O_3_. In addition, the percentage of insoluble residue (I.R.) was determined by an acid HCl test in a solution of hydrochloric acid. The calculation of the contents of the aforementioned oxides, as well as the (I.R.), was carried out using Equations (1)–(7), which are presented in the following paragraphs.

Determination of pure SiO_2_:

(1) $${\mathrm{SiO}}_{2\mathrm{Pure}}=\frac{M_{\mathrm{DE}}-M_{D}}{M_{\mathrm{TS}}}\times 100$$

where M_DE_: mass of the sample obtained by double evaporation; M_D_: determined mass (gm); M_TS_: mass of the sample tested (gm).

Determination of soluble SiO_2_:

(2) $${\mathrm{SiO}}_{2\mathrm{Soluble}}=\frac{500\times M_{\mathrm{CS}}\times 100}{20\times 1000\times M_{\mathrm{TS}}}=2,5\frac{M_{\mathrm{CS}}}{M_{\mathrm{TS}}}$$

where M_CS_: silica concentration in the solution (mg of SiO_2_, in 100 mL); M_TS_: initial mass of the sample tested (gm).

Total SiO_2_ is the result of the sum of the pure silica content and the soluble silica content (1) and (2). 

Determination of Al_2_O_3_:

(3) $${\mathrm{Al}}_{2}O_{3}=\frac{0.03\times 101.961\times 500\times V_{\mathrm{VS}}\times F_{\mathrm{DF}}}{2\times 1000\times 100\times M_{\mathrm{TS}}}\times 100=0.7647\times \frac{V_{\mathrm{VS}}\times F_{\mathrm{DF}}}{M_{\mathrm{TS}}}$$

where Vvs: volume of the dissolution of ethylenediaminetetraacetic acid (EDTA) (~ 0.03 mol/L) consumed in the titration (mL) plus the volume VEX, in mL; F_DF_: EDTA dissolution factor 0.03 mol/L; M_TS_: initial mass of the sample tested (gm); VEX: excess volume in the dissolution of EDTA.

Determination of Fe_2_O_3_:

(4) $${\mathrm{Fe}}_{2}O_{3}=\frac{0.03\times 159.692\times 500\times V_{\mathrm{vs}}\times F_{D}}{2\times 1000\times 100\times M_{\mathrm{TS}}}\times 100=1.1977\times \frac{V_{\mathrm{VS}}\times M_{\mathrm{TS}}}{M_{\mathrm{TS}}}$$

where V_VS_: volume of the dissolution of EDTA (~0.03 mol/L) consumed in the titration (mL); F_D_: dissolution factor EDTA (~0.03 mol/L); M_TS_: mass of the sample tested (gm).

Determination of the CaO:

(5) $$\mathrm{CaO}=\frac{0.03\times 56.08\times 500\times V_{\mathrm{ED}}\times F_{G}}{1000\times 25\times M_{\mathrm{TS}}}\times 100=3.3648\frac{V_{\mathrm{ED}}\times F_{G}}{M_{\mathrm{TS}}}$$

where V_ED_: EGTA (ethyleneglycoltetraacetic acid) dissolution volume (~0.03 mol/L), consumed at titration, in mL; F_G_: dissolution factor EGTA (~0.03 mol/L); M_TS_: mass of the sample tested (gm).

Determination of SO_3_:

(6) $${\mathrm{SO}}_{3}=\frac{M_{\mathrm{BS}}\times 0.343\times 100}{M_{\mathrm{TS}}}=34.3\times \frac{M_{\mathrm{BS}}}{M_{\mathrm{TS}}}$$

where M_TS_: mass of the sample tested (gm); M_BS_: barium sulphate mass (gm).

Determination of the insoluble residue (I.R.):

(7) $$I.R.=\frac{M_{\mathrm{IR}}}{M_{\mathrm{TS}}}\times 100$$

where M_IR_ = mass of calcinated insoluble residue (gm); M_TS_ = mass of the sample tested (gm).

#### 4.2.5. Pozzolanicity Analysis (PA)

The procedures for the chemical test of pozzolanicity were adapted to the UNE-EN 196-5:2011 standard [27]. First, 100 mL of distilled water was heated to 40 °C for one hour. Then, 20 gm of zeolite mixed with cement was added (ratio 75:25%). It was shaken for 20 s. After 8 days, the solution was filtered. The calculation of the concentration of hydroxyl ions [OH^−^] was performed through the following expression [27].

(8) $$\left[{\mathrm{OH}}^{-}\right]=\frac{1000\times 0.1\times V_{3}\times f_{2}}{50}=2\times V_{3}\times f_{2}$$

where [OH^−^]: is the concentration in hydroxyl ions (mmol/L); V_3_: volume of the hydrochloric acid solution (0.1 mol/L); f_2_: the factor of the hydrochloric acid solution (0.1 mol/L).

The concentration of calcium oxide (CaO) was calculated through the following equation [27].

(9) $$\left[\mathrm{CaO}\right]=\frac{1000\times 0.025\times V_{4}\times f_{1}}{50}=2\times V_{4}\times f_{1}$$

where [CaO]: is the concentration in calcium oxide (mmol/L); V_4_: the volume of EDTA solution used in the titration; f_1_: the factor of the EDTA solution.

According to the UNE-EN 196-5:2011 standard [27], the mixture of mordenite–clinoptilolite with cement in distilled water and cement at 40 °C should achieve equilibrium after 8 days.

## 5. Conclusions

The results presented and discussed demonstrate a close genetic and spatial relationship between mordenite and clinoptilolite. Both minerals have been formed as secondary species through a process of devitrification and hydrothermal alteration of the volcanic glass. The chemical composition shows appreciable values of SiO_2_ and Al_2_O_3_, which indicates that the zeolites analysed are undoubtedly good-quality pozzolans for use in the manufacture of pozzolanic cements, without negative impacts on the environment. These cements have the property of resisting the attack of seawater and sulphates, which is beneficial for the Island of Cuba, where the main cities and tourist infrastructures are concentrated on the coast. Lastly, the manufacture of pozzolanic cements is low in cost, which could have a positive impact on the economy of this country, principally in the construction of cheap housing for the population, which is often affected by hurricanes and earthquakes.

## Figures and Tables

**Table 1:** Chemical composition of the zeolite from the area of research (analysis by XRF + A. atomic-sodium).

Sample	SiO_2_	Al_2_O_3_	Fe_2_O_3_	CaO	MgO	K_2_O	Na_2_O	LOI *	Si/Al
ZPH-1	64.72	12.75	1.77	4.09	0.25	2.13	0.22	12.11	4.5
ZPH-2	64.57	12.57	1.32	2.42	0.74	1.65	1.33	14.15	5
ZPH-3	64.94	13.25	1.83	2.34	0.63	2.33	0.68	12.32	4.3
ZPH-4	64.20	11.30	1.40	2.17	0.49	1.77	1.34	14.53	5
ZPH-5	65.02	13.17	1.79	1.95	0.63	2.19	0.51	12.67	4.3

* Loss on ignition.

**Table 2:** Chemical characterisation to determine the pozzolanic quality of the mordenite–clinoptilolite.

%	ZPH-1	ZPH-2	ZPH-3	ZPH-4	ZPH-5	Maximum Allowed Content (%)
Total SiO_2_	64.89	64.08	64.63	64.33	64.98	
MgO	0.79	1.21	0.76	0.55	0.71	<5
Total CaO	3.69	2.29	2.16	2.13	2.03	-
Fe_2_O_3_	1.69	1.34	1.70	1.37	1.62	-
Al_2_O_3_	12.91	12.55	12.99	11.21	13.0	<16
Reactive SiO_2_	60.28	61.02	61.20	60.75	61.13	>25
SO_3_	0.04	0.05	0.03	0.03	0.02	<4
Reactive CaO	1.23	1.14	1.08	1.06	1.01	-
I.R.	4.7	3.90	3.43	4.0	4.0	<5
SiO_2_/(CaO + MgO)	14.50	18.30	22.13	24.0	23.71	>3.5
S_i_O_2_ + Al_2_O_3_ + Fe_2_O_3_	79.49	77.97	79.32	76.91	79.6	>70

## Data Availability

Not applicable.

## Sample Availability

Not available.
